# Formative assessments during COVID-19 pandemic: an observational study on performance and experiences of medical students

**DOI:** 10.12688/mep.19428.2

**Published:** 2023-08-07

**Authors:** Vanessa Lavallard, Bernard Cerutti, Marie-Claude Audétat-Voirol, Barbara Broers, Julia Sader, Annick Galetto-Lacour, Stéphane Hausmann, Georges L. Savoldelli, Mathieu Nendaz, Monica Escher

**Affiliations:** 1 Faculty of Medicine, University of Geneva, Geneva, Switzerland; 2 Unit of Development and Research in Medical Education, Faculty of Medicine, University of Geneva, Geneva, Switzerland; 3 Pediatrics, Gynecology and Obstetrics, Geneva University Hospitals, Geneva, Switzerland; 4 Anesthesia, Pharmacology and Intensive Care, Geneva University Hospitals, Geneva, Switzerland; 5 Division of General Internal Medicine, Geneva University Hospitals, Geneva, Switzerland; 6 Division of Palliative Medicine, Geneva University Hospitals, Geneva, Switzerland

**Keywords:** assessment, medical education, distance education, COVID-19

## Abstract

**Background**: Because of COVID-19, the 2020 written medical examinations were replaced by mandatory formative online assessments. This study aimed to determine students’ performance, self-assessment of performance, and perception about the switch from a summative to a formative approach.

**Methods**: Medical students from year 2 to 5 (n=648) were included. They could repeat each test once or twice. They rated their performance after each attempt and were then given their score. Detailed feedback was given at the end of the session. An online survey determined medical students’ perception about the reorganization of education. Two items concerned the switch from summative to formative assessments

**Results**: Formative assessments involved 2385 examinees totaling 3197 attempts. Among examinees, 30.8% made at least 2 attempts. Scores increased significantly at the second attempt (median 9.4, IQR 10.8), and duration decreased (median -31.0, IQR 48.0). More than half of examinees (54.6%) underestimated their score, female students more often than male. Low performers overestimated, while high performers underestimated their scores. Students approved of the switch to formative assessments. Stress was lessened but motivation for learning decreased.

**Conclusions**: Medical students’ better scores at a second attempt support a benefit of detailed feedback, learning time and re-test opportunity on performance. Decreased learning motivation and a minority of students repeating the formative assessments point to the positive influence of summative assessment on learning.

## List of abbreviations

**MCQ:** Multiple-choice questions

**ECTS:** European Credit Transfer and Accumulation System

**OSCE:** Objective Structured Clinical Examination

## Introduction

The Faculty of Medicine at the University of Geneva, like most medical schools internationally, had to adapt rapidly to the changes brought about by the COVID-19 pandemic, and set up online teaching ( Dost *et al.*, 2020; LeBlanc III, 2022; Stoehr *et al.*, 2021). With regard to assessment, most medical schools decided to cancel summative examinations or to defer them until the end of lockdown ( Jodheea-Jutton, 2021). Others resorted to different approaches to administer summative exams such as online random multiple-choice questions (MCQ) and open-book examination ( Birch & de Wolf, 2020; Pather *et al.*, 2020). In March 2020, the Geneva Faculty of Medicine decided to cancel all the summative oral and written examinations, except for the high-stake selection examination taking place at the end of the first year. The decision was based on educational, ethical, and public health-related considerations. The delay was short to organize valid and reliable distance summative assessments. As medical students were solicited for clinical and logistical work during the health crisis, equity towards students engaged in much needed volunteer activities was a concern.

The main aim of summative assessment is to evaluate whether the required skills and knowledge have been acquired at some definite timepoint. Assessments are usually high-stakes as they are used for promotion decisions. Formative assessment is a process of evaluation and feedback. The objective is to support and accompany the learning process. Formative assessments are typically low-stakes, ongoing, and seek to determine where the students are in their learning, what they need to improve and how they can do it ( Dixson & Worrell, 2016; Dolin *et al.*, 2018). Any kind of assessment however contributes to and drives learning ( Crossley *et al.*, 2002; Schuwirth & Van der Vleuten, 2011; van der Vleuten & Schuwirth, 2005). Formative assessments are described as having a positive impact on learning in medical education ( Dunlosky *et al.*, 2013; Roediger & Karpicke, 2006), especially when they are associated with a detailed feed-back ( Agius & Wilkinson, 2014). Despite the constraints associated with the COVID-19 pandemic, maintaining some form of assessment seemed crucial to evaluate students’ competencies, to stimulate and support their learning, and as a benchmark for students to measure their progress. Therefore, the summative examinations at the Geneva Faculty of Medicine were replaced with mandatory formative online assessments (i.e. students were required to participate, but not required to pass).

Assessment however contributes to and drives learning ( Crossley *et al.*, 2002; Schuwirth & Van der Vleuten, 2011; van der Vleuten & Schuwirth, 2005). Despite the constraints associated with the COVID-19 pandemic, maintaining some form of assessment is crucial to evaluate students’ competencies, to stimulate and support their learning, and as a benchmark for students to measure their progress. Formative assessments are described as having a positive impact on learning in medical education ( Dunlosky *et al.*, 2013; Roediger & Karpicke, 2006), especially when they are associated with a detailed feed-back ( Agius & Wilkinson, 2014). Therefore, the summative examinations at our Faculty were replaced with mandatory formative online assessments (i.e. students were required to participate, but not required to pass).

There is a paucity of data regarding students’ performance and perception when summative examinations were substituted with formative assessments in response to COVID-19. In one study, first-year medical students felt that formative online tests were useful for their learning ( Snekalatha *et al.*, 2021). The aim of this study was to determine medical students’ performance at the 2020 formative assessments, and their perception about the switch from a summative to a formative approach. In June 2020, an online survey was conducted among all the medical students from year 2 to 6 to determine how students were organizing their activities, and the impact of the pandemic on their personal life, training and professional identity. ( Wurth *et al*., 2021) The current study shares the same medical students pool and focuses on formative assessment during COVID-19 pandemic.

## Methods

We conducted an observational study including all the medical students from year 2 to 5 (n=648) of the Geneva Faculty of Medicine whose 2020 written summative exams were switched to formative assessments.

At the Geneva Faculty of Medicine, the 6-year curriculum is divided into Bachelor years (1 to 3) and Master years (4 to 6). Year 6 is a clinical year. After completing their clinical clerkships, students sit the Swiss federal licensing exam. The medical students from year 2 to 5 were concerned by the online formative assessments and were included in the study ( Table 1). Participation was mandatory to obtain the ECTS (European Credit Transfer and Accumulation System) credits and validate the current year.

**Table 1.** Medical students’ demographic information (year of study and gender).

		Year of study			
		Bachelor 2 (n= 183)	Bachelor 3 (n= 150)	Master 1 (n= 157)	Master 2 (n= 158)
**Gender**	**Female (n= 396)**	117	96	91	92
	**Male (n= 252)**	66	54	66	66

All the written MCQ exams were maintained as formative assessments and conducted online on a secured platform of the Faculty of Medicine (Moodle Platform version 2.1.0). In Bachelor years, 5 written formative assessments were held for respiration, osteo-articular system, infectious diseases, integration, and community dimensions. In Master years, 10 written formative assessments were held for surgery, internal medicine, primary care, paediatrics, gynaecology and obstetrics, psychiatry, radiology, pathology, ophthalmology, and emergency medicine and intensive care. We collected the data for all the written formative assessments. Objective Structured Clinical Examinations (OSCEs) and oral examinations were cancelled, or adapted and conducted at distance, and were not included in the present study.

In June 2020, we conducted an online anonymous cross-sectional survey ( Wurth *et al*., 2021) of all the medical students from year 2 to 6 to determine their perception of the impact COVID-19 had on the curriculum. Data about assessment for students year 2 to 5 were included in this analysis.

## Ethics approval and consent to participate

The study was submitted in February 2022 to the University Committee for Ethical Research at the Geneva University (CUREG) which waived the need for review because the study was an analysis of anonymized collected data.

All methods were carried out in accordance with relevant guidelines and regulations.

Written informed consent was obtained from participants in the survey. A general written informed consent is given by all the medical students from year 2 to 6 to use the anonymized results of assessments for research and quality purposes.

### Formative assessments

To reach our aim - i.e., support students’ drive for learning through assessment - we relied on a combination of incentives and formal requirements. The tests took place during the period of time originally devoted to the examination session. They were similar to the usual exams concerning content and format. The tests had the same length (number of questions, examination time) and the content was chosen according to the blueprint of each discipline. The questions were not specifically developed for the formative tests, but they were taken from the pool used for the summative examinations. Previous exams were used in different disciplines – i.e., in Bachelor years respiration, osteo-articular, and infectious diseases units and in Master years psychiatry, gynaecology-obstetrics, internal medicine, primary care medicine, paediatrics, ophthalmology, radiology and pathology. So, the formative tests offered to the students were valid and reliable.

Once students had begun a test, they had a limited amount of time to complete it, identical to the usual examination time. They had to answer the questions sequentially, i.e. they could not go backwards and modify the answers of previously answered questions. After completion of an attempt, students were given their scores. The same set of questions was used for each attempt. At the end of the whole assessment session, they were given detailed feed-back for each test. They had access to all the questions, the correct answers, and their own answers. Master students were also provided with the following summary: their own score for each attempt, and for comparison the lowest and the highest observed scores, and the 10%, 25%, 50%, 75%, 90% quantiles.

In Bachelor years, three written formative tests, namely respiration, osteo-articular, and infectious diseases, were opened for two periods of 24 hours separated by a 24-hour break. Only one attempt was authorized per period, for a total of two attempts. Two disciplines, namely integration and community dimensions, were organized according to a different modality: three attempts were authorized within a time span of 5 days.

In Master years, ten written formative assessments were held and students could access the test for each discipline within a predetermined time span of 72 hours, with a maximum of 3 attempts allowed.

Before each test, students were given recommendations about how to benefit from the formative assessments. They were instructed to take the test in conditions favouring concentration, to answer the questions on their own, to look for the information that would have been missing after each attempt, and to use the detailed feedback to supplement their learning based on identified gaps.

Students were asked to complete a short questionnaire at the end of each formative test. The questionnaire included one item about self-assessment of performance (“I think my score in this formative test is: 1) less than 20%, 2) between 20 and 40%, 3) between 40 and 65%, 4) between 65 and 80%, or 5) more than 80%”) and one item about their wish for support in learning (“I would like to have feed-back or advice about how to study”). The choice of answers was: 1) “not at this stage” 2) “maybe” 3) “yes”

### Survey

An online survey (evasys software, version 8.2) was conducted among all the medical students from year 2 to 6 (N=803) in June 2020 ( Wurth *et al.*, 2021). The aim was to explore the students’ perception about the reorganization of teaching and the switch from summative to formative assessments. In this previous study, the data concerning formative assessments were not analysed. Two items concerned assessment: 1) “Most examinations have been reorganized since March and switched from summative exams (pass/fail with grades) to mandatory formative assessments. I think this was a good decision (1 strongly disagree, 4 strongly agree)”; 2) “What impact did the switch from summative to formative assessments have on you? (free text)”

Medical students from year 2 to 5 (n=648) were affected by the switch to formative assessments and participated in this research. We used a conventional content analysis approach to analyse item 2. Two researchers (VL, ME) independently read the participants’ comments. A list of seven codes was developed, and then independently applied to all the comments. Coding discrepancies were identified and resolved by consensus.

### Reflexivity

VL is a biologist and responsible for the Bachelor examinations. ME is a physician and responsible for the Master examinations at the Geneva Faculty of Medicine. ME conducted or was involved in previous qualitative research projects. VL and ME had no direct contact with the students apart from sending written information related to the taking of the formative assessments. They organized the formative assessments and ensured that they ran smoothly.

### Statistical analysis

Fifteen formative assessments were considered for the analyses: five Bachelor exams and ten Master exams.

For each attempt, the test scores were computed by dividing the number of points of each examinee by the maximum number of points achievable (e.g., 50 if half of the points were achieved). The scores for each exam were standardized (mean 100 and standard deviation 10). The duration of an attempt was defined as the interval between the beginning of the attempt and either the final validation of the answers by the examinee or the automatic validation of the answers by the program whenever the time allowed was elapsed. Since the duration of the formative assessments differed among the disciplines, it was standardized (mean 0 and standard deviation 10) when the association between the scores and the duration of each attempt was investigated.

We used chi-square tests to investigate the association between categorical variables and analysis of variance to investigate the association between a numerical variable and a categorical variable. We used linear mixed effect model to investigate the association between the standardized scores and standardized duration as well as gender (fixed effects), and students (random factor).

No data imputation method was used to estimate missing values. Tests were used with a Type I error rate of 0.05. All analyses were made using R software, version 4.1.1 (The R Foundation for Statistical Computing, Vienna, Austria).

## Results

### Formative assessments

***Number of attempts and duration*.** Fifteen tests (5 in Bachelor years and 10 in Master years) involved 2385 examinees totalling 3197 attempts, for a total of 648 students (Table 1). No students was excluded from analysis. The average number of attempts was 1.34: 1651 (69.2%) examinees made a single attempt, 656 (27.5%) made two attempts, and 78 (3.3%) made three attempts. The proportion of examinees making a single attempt was higher among Bachelor students when compared with Master students (75.9% vs. 65.8%; p<.0001), with no evidence of a difference between gender (p=.22).

**Figure 1.**
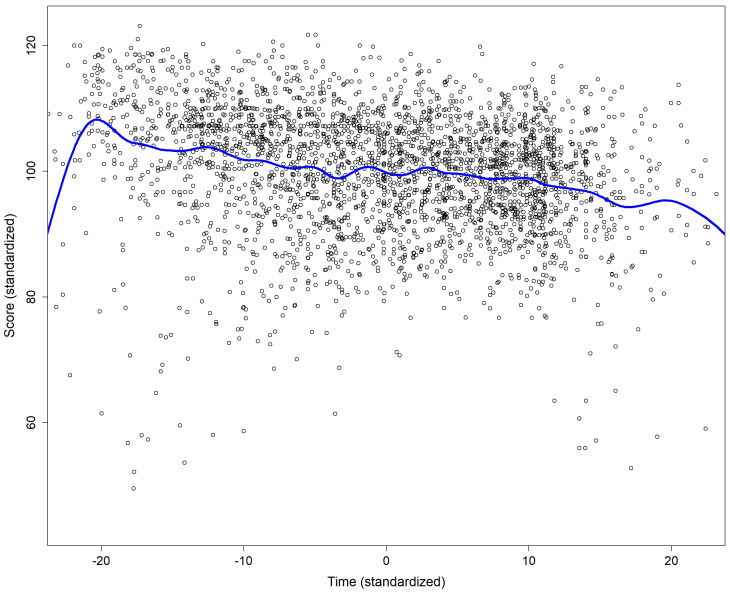
Association between the score and the duration of the attempt. The continuous blue line was obtain using smoothing spline fitting.

For Master students the average interval between the first and the second attempt was 16 hours (median 7 hours), and 18 hours between the second and the third attempt (median 14 hours). Apart from a few outliers (very short or very long durations) there was a trend of a negative association between the score and the duration of the attempt (p<.0001 for the t value associated with the estimate of the slope; Figure 1).

**Figure 2.**
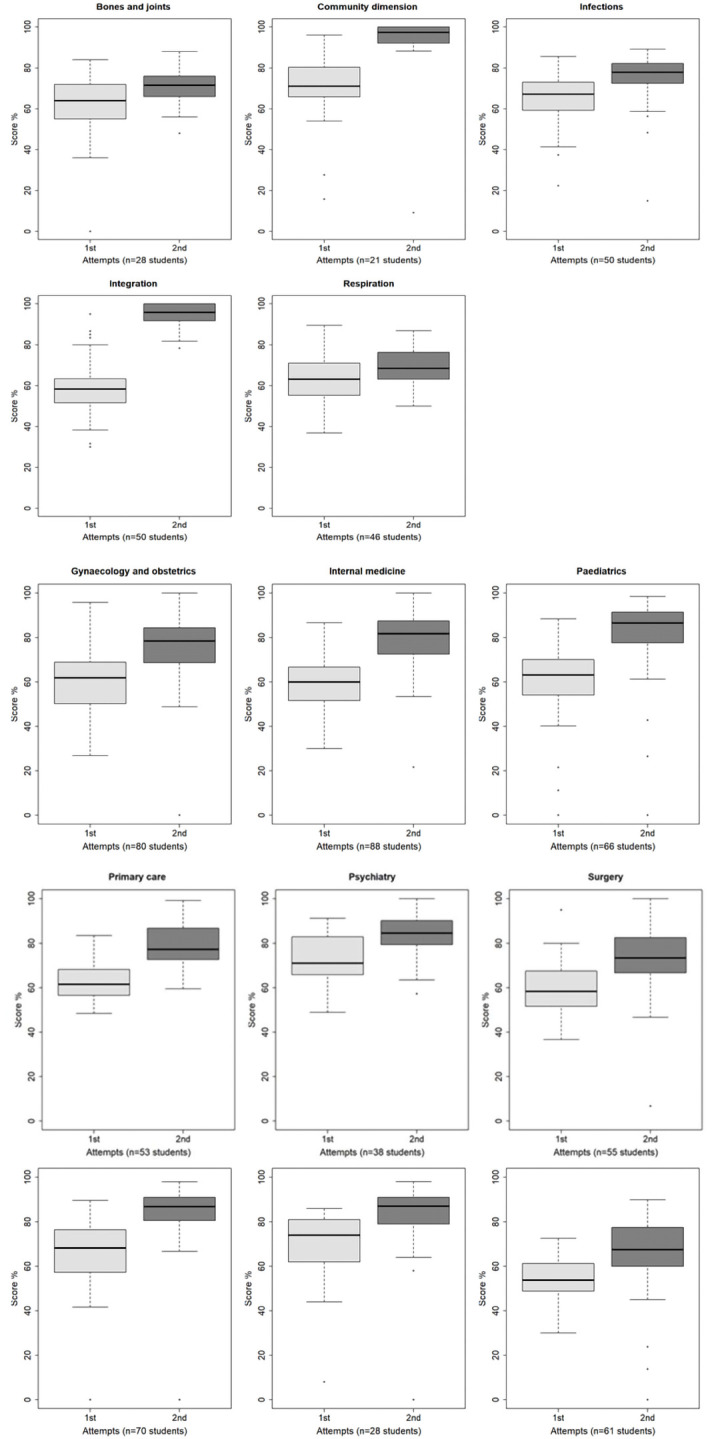
Boxplot of the scores at first and second attempt (subgroup of students who made several attempts).

Figure 2 shows that the average score at the first attempt was higher for the students making a single attempt compared to those making 2 or more attempts for 10 tests: community dimension (p<.0001), integration (p<.0001), surgery (p<.0001), gynaecology and obstetrics (p<.0001), internal medicine (p<.0001), primary care (p=.0018), ophthalmology (p=.0425), paediatrics (p<.0001), psychiatry (p=.0009), emergency and intensive care (p=.0026). There was no significant difference for four tests: bones and joints (p=.0952), respiration (p=.2450), infections (p=.0662), and pathology (p=.6200). No student made a second attempt for the test in radiology.

***Subgroup of examinees with several attempts*.** For the subgroup of examinees who made at least two attempts (734 examinees; 30.8%), the average increase in score between the first and the second attempt was +10.5 (from -41.6 to +51.7; median 9.4, IQR 10.8). The average decrease in duration of the attempt was 33.1 min (from -123.0 to +53.0; median -31.0, IQR 48.0).

The highest score was most often achieved at the second attempt (85.4%), the other situations being equally distributed between the first (7.5%) and the third attempt (7.1%) ( Figure 2). In the most common situation (529 examinees, 72.1%) the highest score was reached at the second attempt and the longest attempt duration was the first.

### Self-assessment and score

**Figure 3.**
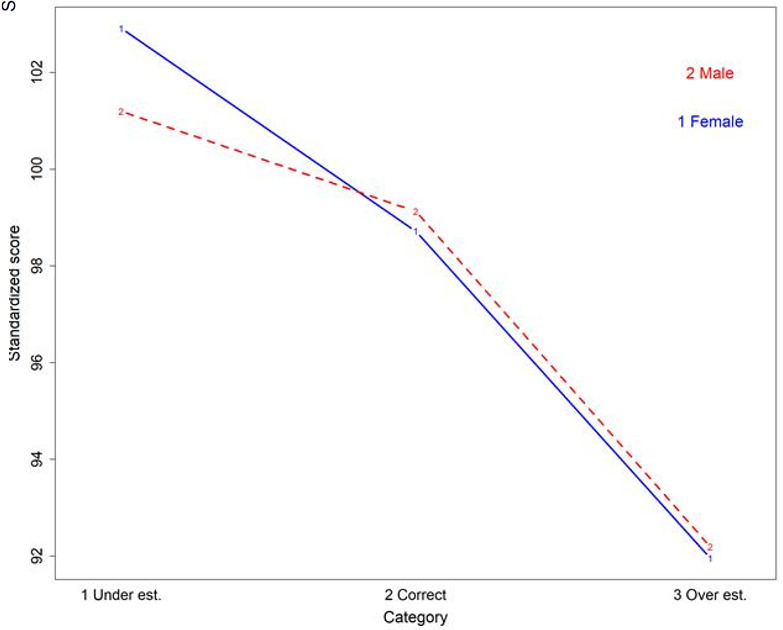
Relationship between standardized scores, self-assessment and gender.

Examinees self-assessed their scores for 2998 attempts (93.8%). More than half of them (54.6%) underestimated their score. They correctly estimated their score in 40.7% of cases, and they overestimated their score for a minority of attempts (4.7%). More female than male students underestimated their scores (60.7% vs. 45.5%; p<0.0001). Students who performed best were more likely to underestimate their scores, while low performers overestimated their scores (p<0.0001). More high performing female students underestimated their scores compared with their male counterparts (p=0.0066) ( Figure 3). Bachelor students were less likely to underestimate their score than Master students (48.0% vs 57.8%; p<.0001).

### Wish for support in learning

**Figure 4.**
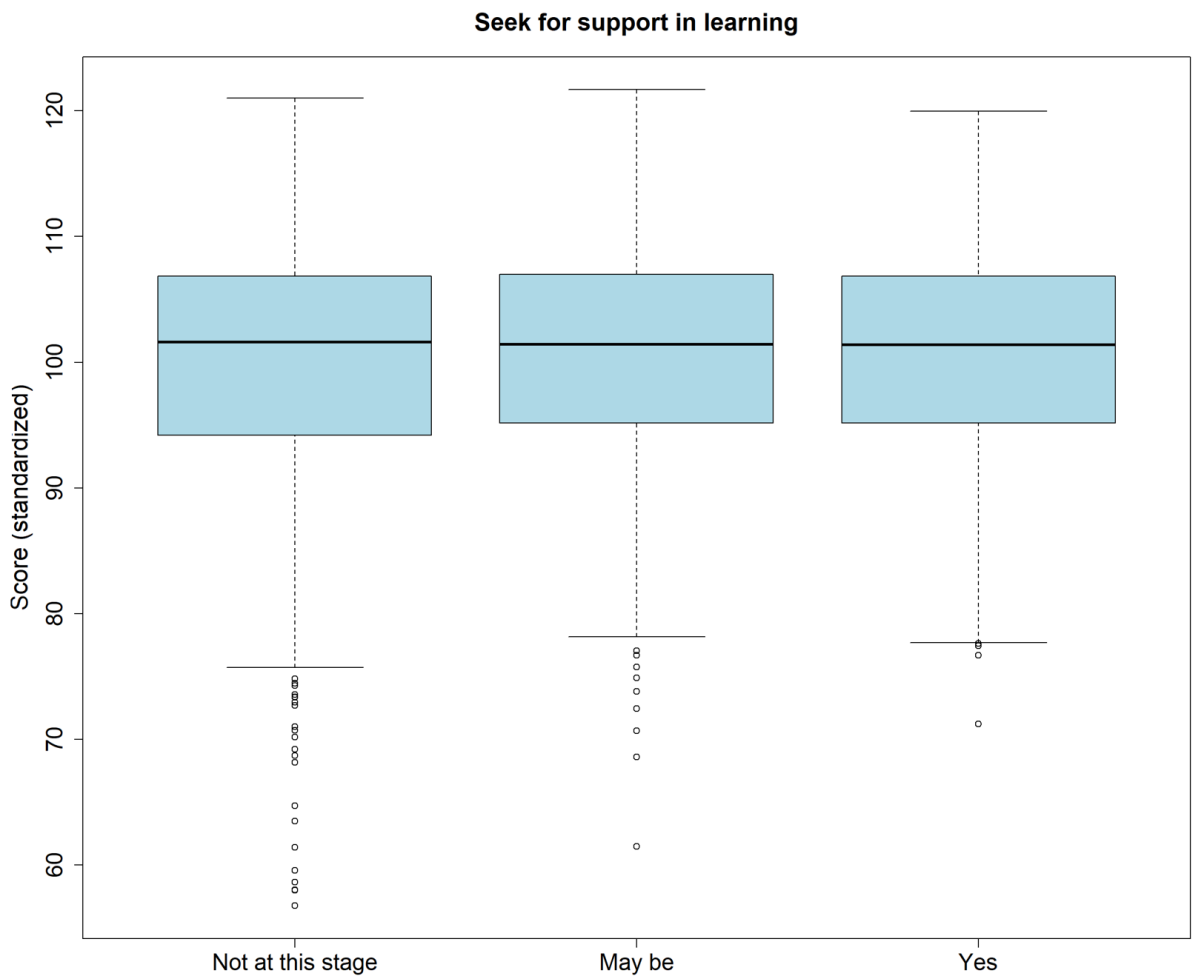
Boxplot of the standardized scores split by indication regarding the perceived need for support in learning.

Across all attempts, 1388 examinees (43.4%) indicated that they had no wish for support at this stage, 900 (28.1%) that they may like to have support, and 500 (15.6%) that they did wish for support. A minority of examinees (n=409; 12.8%) did not respond. There was no association between the wish for support and self-assessment of score (p=.365), or the actual normalized score (p=.109; Figure 4).

### Survey results

Out of 648 medical students, 390 (60.2%) students answered the questions about assessment. The majority (87.9%) strongly agreed or agreed with the switch from summative assessments to mandatory formative assessments. A minority (11.0%) disagreed or strongly disagreed, and 1.0% had no opinion.

**Figure 5.**
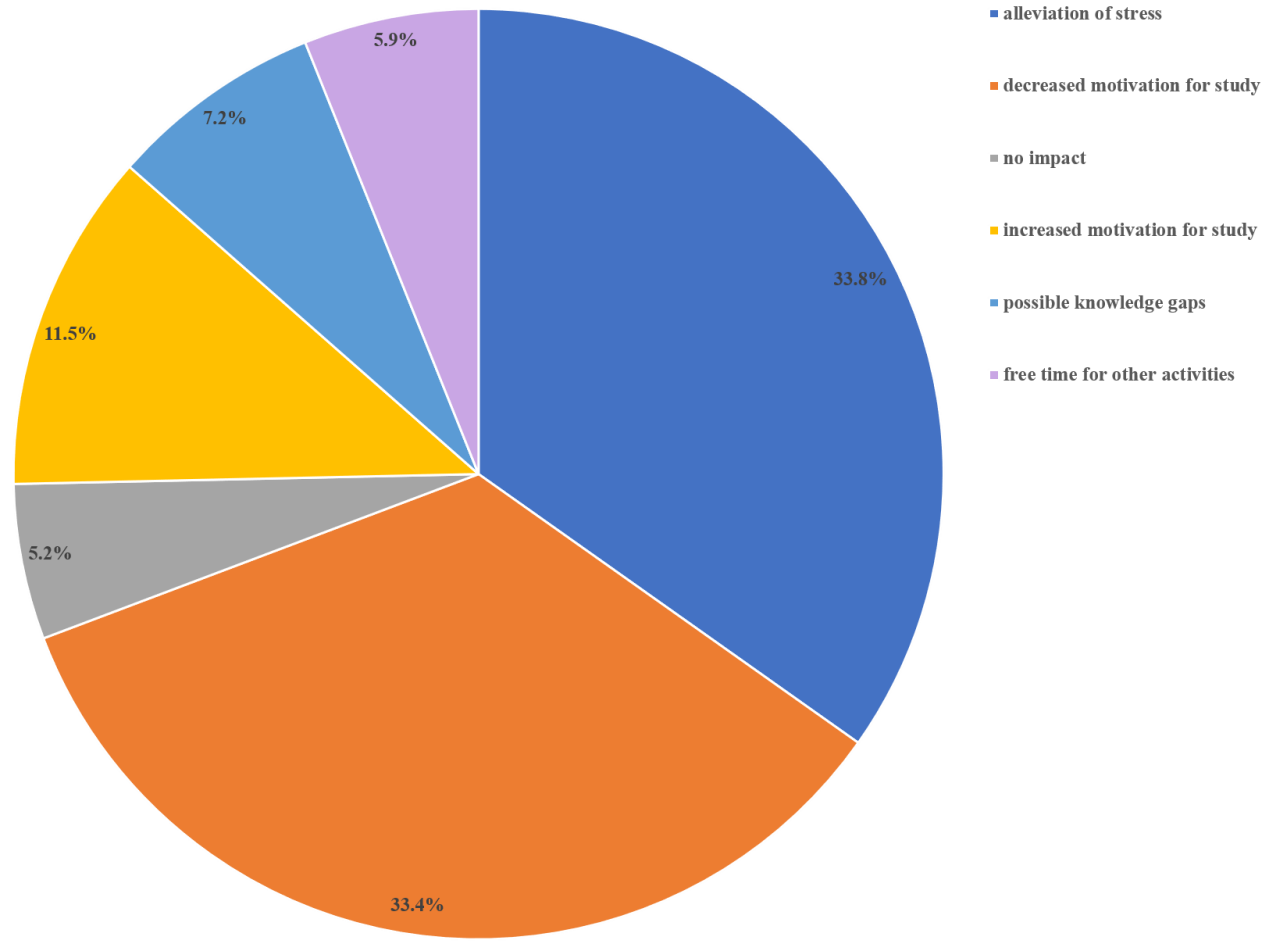
Survey results: “What impact did the switch from summative to formative assessments have on you? (Free text)”.

Among the respondents, 305 (78.2%) answered the open-ended question about the impact of the switch to formative assessments. The responses are in French and have been translated into English. We did not code 57 comments (19.6%) either because they were made by 6 ^th^-year students or because they were not related to the question. Six themes emerged: 1) alleviation of stress, 2) decreased motivation for study, 3) no impact, 4) increased motivation for study, 5) fear of gaps in knowledge, and 6) free time for other activities ( Figure 5). Representative quotes for each theme are presented in Table 2. The most common perceptions were relief (33.8%), and decreased motivation for study (33.4%). The two feelings were often associated in a same comment: *“Less stress, but consequently more difficult to get motivated and study”*. A minority of students (11.5%) experienced enhanced motivation: *“I enjoyed learning not for an exam, but to increase my own knowledge, out of the desire to understand the processes”*. Some students wondered about potential gaps in knowledge and how it could impact their future professional curriculum. Others appreciated the extra free time, which they used for other activities, especially volunteer work for the COVID-19 health crisis.

**Table 2.** Impact of the switch from summative to formative assessments as perceived by medical students year 2 to 5 (n=305).

Themes	Representative quotes *
Alleviation of stress	*Less pressure* *Relief* *Much less stress, healthier learning conditions*
Decreased motivation for study	*I feel less involved in my learning, it comes as a surprise because I thought I was conscientious* * enough in order not to lose in motivation* *Less motivation for learning and studying long hours* *Much more difficult to stay motivated*
No impact	*No impact* *No change. I studied as if it was a real exam.*
Increased motivation for study	*More motivated to study. More time to gain in-depth knowledge.* *Renewed motivation. I again enjoyed learn new things, for the future, and not just for an exam.* *I learned more out of curiosity and pleasure, and less according to the learning objectives.*
Fear of gaps in knowledge	*Anxiety about going on with my studies, without any evidence of my competencies and capacity* * to pass to the next level.* *Less involvement in studying, less knowledge acquired* *All our 4 ^th^ year exams cancelled, hence fear for the Federal Licensing Exam*
Free time for other activities	*Since the assessments were not summative, I could engage in volunteer work at the hospital.* *It allowed me to participate a little more in the health crisis.* *I have free time for other activities, it makes me happy.*

*the original responses were in French, and were translated for the manuscript

## Discussion

In this study we investigated medical students’ performance and perception after summative examinations were replaced by mandatory formative assessments.

For each test, 2 and 3 attempts were authorized in a predefined time frame for Bachelor and Master students, respectively. Among the 30.8% of examinees who repeated the test, the average score increased, and the average duration of the attempt decreased, which supports a benefit of the combination of detailed feedback, learning time, and re-test opportunity. Taken together with the time duration between two attempts, it suggests that the students who repeated the test studied the learning contents. This is consistent with previous findings about formative assessments during the COVID-19 pandemic where many students (72.2%) indicated that feedback, given after the tests, motivated them to study ( Snekalatha *et al*., 2021). Students who repeated a test were more often in Master than in Bachelor years. One reason for the difference could be the subject matter. In the survey, Master students felt motivated to study topics they considered clinically useful, and Bachelor exams mainly evaluate fundamental knowledge.

It was disappointing that a majority of students – 3 out of 4 in Bachelor years and 2 out of 3 in Master years - did not repeat the test. Although their average score at the first attempt was better than the score of the other students, they missed a learning opportunity. Other studies showed that personal study coupled with a second test improves the anchoring of knowledge and the ability to use it ( Agarwal *et al.*, 2012) ( Roediger & Butler, 2011). This retrieval effect can persist in time and knowledge retrieval is an important aspect of clinical work.

Students’ self-assessment of scores were sub-optimal since less than half of the examinees’ estimations were correct. High-performing students underestimated their scores, while poor performers overestimated them. Female students and students in Master years underestimated their scores to a greater extent than male students and students in Bachelor years, respectively. Inaccuracy in self-assessment in medical students and a difference between female and male students have been observed ( Blanch-Hartigan, 2011; Gordon, 1991). Students can be inaccurate due to the complexity of self-assessment per se and the difficulty in identifying gaps in a specific domain ( Eva & Regehr, 2005; Regehr & Eva, 2006). Overestimation by poor performers and underestimation by good performers was described ( Colthart *et al.*, 2008). Explanations can be a regression to the mean and a lack of the cognitive skills needed for accurate self-assessment in the less competent individuals. Female students tend to be more anxious and less confident than their male counterparts ( Blanch *et al.*, 2008; Colbert-Getz *et al.*, 2013), traits that can be related to a marked underestimation in high performers. The difference can also be related to gender bias. In the clinical vignettes used for teaching at the Geneva Faculty of Medicine gender professional roles tend to be stereotypes, e.g. physicians are more often male ( Arsever *et al.*, 2023). To depict women in leading positions would strengthen the image of women as competent professionals. The assessed subject matter rather than a difference in self-assessment capacity could explain the difference we found between Bachelor and Master students. Master students were asked to evaluate their performance in clinical reasoning whereas Bachelor students evaluated their competence in fundamental knowledge. Students may be more familiar with this kind of content and therefore more capable of assessing their performance.

The proportion of examinees who wished for support and those who did not was similar, and wish for support was not associated with self-assessment of performance or the actual scores. De Jong *et al.* found a relationship between feedback-seeking behaviour and performance: high-performing students were more motivated and self-determined to seek feedback compared to low-performing students ( de Jong *et al.*, 2017). The study setting was different from ours since it included veterinary medicine students in their final year who underwent a summative evaluation at the workplace. The medical students of the Geneva Faculty of Medicine were in their preclinical and clinical years and assessment was formative. Students had extensive online feedback on their performance and were offered support for learning. They seemed to find this opportunity moderately appealing, which is somewhat surprising. Because of the sanitary measures requested by the COVID-19 epidemic, students were isolated and could not benefit from the usual learning opportunities and motivation between peers. They actually reported in the survey to worry about potential knowledge gaps.

Medical students were supportive of the switch from summative to formative assessments. The main reason was an alleviation of stress, but participants also mentioned the opportunity it gave them to volunteer for COVID-19-related activities. A frequently reported drawback was a decreased motivation to study despite the mandatory character of the formative assessments, the authenticity of the tests, and the extensive feedback given. Some students enjoyed the freedom to choose what they learned. A matter of concern is the way they discarded knowledge pertaining to basic sciences to focus on subjects they deemed useful for clinical work. A role of teachers as experts is to guide students’ building up of knowledge by providing relevant learning content. Students’ ability of correctly selecting what matters for knowledge scaffolding is questionable. The understanding of biological and physiopathological mechanisms is essential in clinical work: it helps relate various signs and symptoms, and disease manifestations with treatment choices. The risk of knowledge gaps in basic sciences is faulty clinical reasoning ( Castillo *et al.*, 2018; Kulasegaram *et al.*, 2017).

Our findings underscore the need for formative assessment to be part of a larger assessment system (programmatic assessment) to motivate and benefit medical students best. The role of scholar defined in the CanMEDS ( https://www.royalcollege.ca/ca/en/canmeds/canmeds-framework.html) or PROFILES ( https://www.profilesmed.ch) frameworks requires lifelong learning and a planned approach to learning from physicians. In a competency-based curriculum, students must be supported to acquire the expected autonomy in self-improvement. Regular self-evaluation of scores at formative and summative assessments could help medical students better estimate their competences. Overestimation of performance is a marker for potentially less competent students. Recurrent overestimations could be used to identify medical students in need of support in learning.

This study has several limitations and strengths. We do not have any information about the learning behaviour of the students who made only one attempt. They may have studied after the test to fill knowledge gaps and have benefited from the feedback offered. The participation in formative assessments was mandatory, so we do not know how the students would have behaved if the assessments had been optional. However, we observed a low participation rate in a couple of early formative assessments which were kept optional because of short decisional and organizational timelines. The formative assessments offered to medical students of the Geneva Faculty of Medecine were robust: the questions were taken from the pool used for the summative examinations, students were given detailed feedback, and they had the opportunity to do the test again and monitor their progress.

## Conclusion

Medical students in the Geneva Faculty of Medicine welcomed the switch from summative to formative assessments during the COVID-19 pandemic. A drawback was decreased motivation in learning. Despite formal requirements, thoughtful test organization and detailed feedback, a sizeable majority of students did not take the opportunity of repeated testing. Students who did take the test more than once significantly improved their performance.

## Consent to publish

The authors confirm that there is no information which can potentially identify a participant.

## Data Availability

figshare: Formative assessments during COVID-19 pandemic: performance and experiences of medical students: https://doi.org/10.6084/m9.figshare.21755882 ( Lavallard *et al*., 2022) This project contains the following underlying data: FormativeAssessmentCOVID.csv (this file contains different the raw quantitative data for variables such as score, duration of attempt, gender, year) SurveyCOVID.csv (this file contains the student’s comments) Please note that the responses to the qualitative survey questions are in French. Specific quotes or themes can be translated to English on request. figshare: Formative assessments during COVID-19 pandemic: performance and experiences of medical students: https://doi.org/10.6084/m9.figshare.21755882 ( Lavallard *et al*., 2022) This project contains the following extended data: README Formative Assessment COVID.txt README Survey COVID.txt Supplementary data.png Data are available under the terms of the Creative Commons Attribution 4.0 International license (CC-BY 4.0).
