# Supplementary material for: Formative assessments during COVID-19 pandemic: an observational study on performance and experiences of medical students
Source: MedEdPublish (2016). 2023 Dec 1;13:7. Originally published 2023 Jan 30. [Version 3] doi: 10.12688/mep.19428.3 (PMC10628360; doi:10.12688/mep.19428.3)
Supplement: Peer review history

# Reviewer report: Reviewer response for version 3

**DOI:** 10.21956/mep.21487.r35467

**Published:** 2023-12-14

**Authors:** Mohamad Nurman Yaman

**Affiliations:** 1 Department of Medical Education, Universiti Kebangsaan Malaysia, Bangi, Selangor, Malaysia

I have no further comments to make. Thank you

Have any limitations of the research been acknowledged?

Yes

Is the work clearly and accurately presented and does it cite the current literature?

Yes

If applicable, is the statistical analysis and its interpretation appropriate?

Partly

Are all the source data underlying the results available to ensure full reproducibility?

Partly

Is the study design appropriate and is the work technically sound?

Yes

Are the conclusions drawn adequately supported by the results?

Partly

Are sufficient details of methods and analysis provided to allow replication by others?

Partly

Reviewer Expertise:

Medical Education, Assessment, Student Selection, Curriculum Development and Review and Programme Accreditation

I confirm that I have read this submission and believe that I have an appropriate level of expertise to confirm that it is of an acceptable scientific standard.

# Reviewer report: Reviewer response for version 2

**DOI:** 10.21956/mep.21158.r35018

**Published:** 2023-11-06

**Authors:** Mohamad Nurman Yaman

**Affiliations:** 1 Department of Medical Education, Universiti Kebangsaan Malaysia, Bangi, Selangor, Malaysia

Thank you for the invitation to review the paper “Formative assessments during COVID-19 pandemic: an observational study on performance and experiences of medical students”. It is an interesting assessment topic. Generally the authors have responded to the input by previous reviewers but the write-up probably need major revision before being accepted for publication.

**Introduction**

 1 st para- adequate 2 nd para- the authors tried to explain summative assessment and formative assessment as general terms (giving references to other journals). Suggestion is to include the actual definition/operational of summative and formative assessments used by the Faculty of Medicine, University of Geneva, so that readers able to appreciate the actual summative and formative assessment done by the faculty during this study period. These were partly explained in the Methods section and took a while to understand how the assessments were being conducted at the faculty.

 3 rd para- this paragraph stated that there are 1) students’ performance and 2) perception of switching from summative assessment to formative assessment. Unfortunately, they don’t match with the Abstract as in the abstract, the aims are to determine 1) students’ performance, 2) self-assessment of performance and 3) perception of students towards the switching from summative to formative approach.

 The following sentences after the aim of this study should be omitted as no comparison between June 2020 study with current study were being evaluated. The terms ‘shares the same medical students pool and focuses on formative assessment….” made more confusion and leading towards one same study data sliced into two publications.

**Methods**

 1 st para- this paragraph is confusing and not sure what information is being delivered eg number 2020 is representing the batch of students or the year or something else.

 2 nd & 3rd and subsequent paragraphs- suggest rewriting this paragraph to explain the curriculum and its content in the beginning (the disciplines) and then explain the assessment approach taken (pre-covid era). Then explain what the changes are done and how it is conducted during covid19.

 Anything less than 10 should be written in words, not numbers.

 Suggest also to omit the June 2020 study as it makes things more confusing.

 Ethics paragraph should be located after the methods, not in the middle of methods section

**Formative Assessment **section to be rewritten together with the paragraph before the ethics based on the earlier suggestion methods layout (intro to assessment in the faculty, pre-covid assessment (summative), formative assessment, the conduct of the assessment).

 Suggest separating Bachelor level and Master level as both cannot be compared to each other. The comparison should be Bachelor Summative vs Bachelor Formative and Master Summative vs Master Summative.

 Overall generalisation should not be done as Bachelor and Master students are not homogenous.

 How do the questions be selected? Having given the same questions used by previous batches/years, it basically will nullify the assessment as the senior students are able to pass their exams questions to their juniors. What are the ways taken by the faculty to secure theses exam question? (Questions selected based on difficulty degrees from a pool of many questions? Are the questions vetted for covid batches? Or any other ways?)

 The last paragraph of Formative Assessment section stated more towards self-perceptions of their performance rather that actual students’ performance (based on grades or marks).

**Survey**

 1 st para- suggest omitting as study done in June 2020 make lots of confusion to the current study that are being focused (eg N=803 vs n=648).

 This section should state how the survey was conducted and what are the items in the survey/instrument/questionnaire. Has this instrument been validated?

**Reflexivity** or Roles by Authors should be located at the end, rather than be a content in the main study text.

**Statistical analysis **- OK

**Results**

 Ideally the result should answer the aims of the study which are to determine 1) students’ performance, 2) self-assessment of performance and 3) perception of students towards the switching from summative to formative approach. This section ideally presented using table for the demographic data.

 Please do include the disciplines during bachelor and master in the table format.

 All the numbers in this section will be best presented in table form, so that comparison between bachelor, masters, discipline, number of attempts can be made adequately, not in narrative form.

 Table 1 should be in Result section.

 My suggestion to review the usage of box plot in Figure 2, how best each discipline be presented and easily understood by the readers.

 Major rephrasing and restructuring of this section are needed so that the readers able to appreciate the process and the flow of result of this study.

 For Figure 5, I believe that bar chart will be better than pie chart as these are free texts and many ideas can be obtained from 1 respondent hence 100% of the pie chart doesn’t represent the whole batch.

 Figure 2 and Table 2 are explaining the same outcomes

**Discussion**

 This section was written not according to the flow or the aims of the study. The best way to present is to match with the flow of the result so that the readers will be able to appreciate the discussion and relate directly with the result section.

 There are numbers and percentages in discussion section that should be stated in result section as the discussion section will explain the nature of multiple attempts by the students.

 Major rephrasing and restructuring of this section are needed so that the readers able to appreciate the flow of discussion of this study.

 For limitation and strength, suggest starting with strength and followed by limitation.

 The author needs to show to the reader why this study is important and can be learnt by others, then followed by the limitations occurred during the study.

**Conclusion**

 The conclusion adequately portrait the summary of the study. Due to the missing students’ performance, the last sentence can’t be made as a conclusion, as the study at present showed self-perception of performance rather than actual performance (as the responses were anonymously recorded).

 Conclusion might need rewriting based on the new writeup of Result and Discussion sections

Have any limitations of the research been acknowledged?

Yes

Is the work clearly and accurately presented and does it cite the current literature?

Yes

If applicable, is the statistical analysis and its interpretation appropriate?

Partly

Are all the source data underlying the results available to ensure full reproducibility?

Partly

Is the study design appropriate and is the work technically sound?

Yes

Are the conclusions drawn adequately supported by the results?

Partly

Are sufficient details of methods and analysis provided to allow replication by others?

Partly

Reviewer Expertise:

Medical Education, Assessment, Student Selection, Curriculum Development and Review and Programme Accreditation

I confirm that I have read this submission and believe that I have an appropriate level of expertise to confirm that it is of an acceptable scientific standard, however I have significant reservations, as outlined above.

# Response

**Published:** 2023-11-30

**Authors:** Vanessa Lavallard

**Affiliations:** Faculty of Medicine of Geneva, Switzerland

Thank you for the invitation to review the paper “Formative assessments during COVID-19 pandemic: an observational study on performance and experiences of medical students”. It is an interesting assessment topic. Generally the authors have responded to the input by previous reviewers but the write-up probably need major revision before being accepted for publication. We thank the reviewer for his feedback and comments on our manuscript" Formative assessments during COVID-19 pandemic: an observational study on performance and experiences of medical students". Please find our answers below.  

**Introduction**  

 1 st para- adequate 2 nd para- the authors tried to explain summative assessment and formative assessment as general terms (giving references to other journals). Suggestion is to include the actual definition/operational of summative and formative assessments used by the Faculty of Medicine, University of Geneva, so that readers able to appreciate the actual summative and formative assessment done by the faculty during this study period. These were partly explained in the Methods section and took a while to understand how the assessments were being conducted at the faculty. The definition of summative and formative assessments, given in the Introduction, is the one used by the Geneva Faculty of Medicine. We explained in the first paragraph of the Introduction that all the summative assessments were cancelled. The summative written assessments were replaced by mandatory formative online assessments (see last sentence of 3 ^rd^ paragraph: “the summative examinations at our Faculty were replaced with mandatory formative online assessments (i.e. students were required to participate, but not required to pass”).    

 3 rd para- this paragraph stated that there are 1) students’ performance and 2) perception of switching from summative assessment to formative assessment. Unfortunately, they don’t match with the Abstract as in the abstract, the aims are to determine 1) students’ performance, 2) self-assessment of performance and 3) perception of students towards the switching from summative to formative approach. The sentence was modified in the last paragraph of the Introduction: “The aim of this study was to determine medical students’ performance at the formative assessments, their self-assessment of performance and their perception about the switch from a summative to a formative approach.”    

 The following sentences after the aim of this study should be omitted as no comparison between June 2020 study with current study were being evaluated. The terms ‘shares the same medical students pool and focuses on formative assessment….” made more confusion and leading towards one same study data sliced into two publications. The current study and the 2020 study are two separate studies with different methods and different aims. The aim of the current study is to determine students’ performance at the formative online assessments, self-assessment of performance, and perception about the switch from summative to formative assessment, whereas the aim of the 2020 study was to determine how students were organizing their activities, and the impact of the pandemic on their personal life, training and professional identity. Since the two studies explore different aspects of the impact of the pandemic, they both include the same cohorts of students. We specified the link between the 2020 study and the current study and the inclusion of the same cohorts of students at the editorial team’s request. It underlines how complementary the two studies are. We put the information in a new paragraph (“In June 2020, etc”) and rephrased the last sentence to make the link clearer (“The current study focused on formative assessment during COVID-19 pandemic in the same cohorts of medical students.”    

**Methods**  

 1 st para- this paragraph is confusing and not sure what information is being delivered eg number 2020 is representing the batch of students or the year or something else. 2020 is the year of the switch from summative to formative assessments. To avoid confusion, we modified the sentence in the first paragraph: “We conducted an observational study including all the medical students from year 2 to 5 (n=648) of the Geneva Faculty of Medicine affected by the switch from written summative exams to formative assessments in 2020.”  

 2 nd & 3rd and subsequent paragraphs- suggest rewriting this paragraph to explain the curriculum and its content in the beginning (the disciplines) and then explain the assessment approach taken (pre-covid era). Then explain what the changes are done and how it is conducted during covid19. The focus of the study is assessment, and not the content of teaching, and we prefer to keep the focus of the manuscript on assessment. At the end of each teaching unit, a written and/or oral summative exam is given to medical students. During the pandemic, the written summative exams were replaced by formative assessments. The details of the formative assessments during the COVID-19 period are given in the section Formative assessments. We modified the beginning of the second paragraph: “At the end of each teaching unit, a written and/or oral summative exam is given to medical students. During the pandemic, all the written MCQ exams were maintained as formative assessments and conducted online on a secured platform of the Faculty of Medicine (Moodle Platform version 2.1.0).”  

 Anything less than 10 should be written in words, not numbers. We replaced “5 written formative assessments” by “five written formative assessments” and “10 written formative assessments” by “ten written formative assessments”.  

 Suggest also to omit the June 2020 study as it makes things more confusing. The data about medical students’ perception of the change from summative to formative assessment were collected as part of the study conducted in June 2020. Therefore, it is necessary to mention the study. We changed the last sentence: “Data about students’ perception of the change from summative to formative assessment were included in this study”   Ethics paragraph should be located after the methods, not in the middle of methods section We moved this paragraph after the conclusion and before the Consent to publish section.          

 Formative Assessment section to be rewritten together with the paragraph before the ethics based on the earlier suggestion methods layout (intro to assessment in the faculty, pre-covid assessment (summative), formative assessment, the conduct of the assessment). All the relevant information is already given in the section, and we prefer to keep it this way since the topic of the paper is “Formative assessments during COVID-19 pandemic”.  

 Suggest separating Bachelor level and Master level as both cannot be compared to each other. The comparison should be Bachelor Summative vs Bachelor Formative and Master Summative vs Master Summative. We agree with the reviewer that Bachelor students and Master students should not be compared and we did not compare them. The circumstances were so different between the summative exams in “normal” years and the formative assessments in the COVID-19 year that we chose not to compare the medical students’ performance in summative vs formative assessments.      

 Overall generalisation should not be done as Bachelor and Master students are not homogenous. The cohorts of Bachelor and Master students are homogenous. The students do all their medical education (from the first year of Bachelor to the last year of Master) in the Geneva Faculty of Medicine. Furthermore, figure 2 shows the results of each single assessment separately.   How do the questions be selected? Having given the same questions used by previous batches/years, it basically will nullify the assessment as the senior students are able to pass their exams questions to their juniors. What are the ways taken by the faculty to secure theses exam question? (Questions selected based on difficulty degrees from a pool of many questions? Are the questions vetted for covid batches? Or any other ways?) As explained in the 1 ^st^ paragraph of the Formative assessment section, formative assessments were similar to the usual exams concerning content and format. The tests had the same length (number of questions, examination time) and the content was chosen according to the blueprint of each discipline. The questions were not specifically developed for the formative tests, but they were taken from the pool used for the summative examinations. The pool of questions is kept on a secured platform and new questions are regularly added. The questions used for a summative exam are not made available to the students, and the ones used for the formative assessments during the pandemic will no longer be used in summative exams.  

 The last paragraph of Formative Assessment section stated more towards self-perceptions of their performance rather that actual students’ performance (based on grades or marks). Figure 3 precisely shows the shape of the association between perceived and actual performance.  

**Survey**  

 1 st para- suggest omitting as study done in June 2020 make lots of confusion to the current study that are being focused (eg N=803 vs n=648). As explained previously, it is necessary - and requested by the editorial team - to cite the 2020 study to explain how the data were collected and what the link between this and our study is. The study done in June 2020 included 803 students, i.e. the students from year 2 to year 6. The current study only includes the students affected by the switch from summative to formative assessments, i.e. 648 students, from year 2 to year 5; since year 6 is a clinical year and students don’t take any exams.   This section should state how the survey was conducted and what are the items in the survey/instrument/questionnaire. Has this instrument been validated? All the details regarding the survey can be found in the article we refer to (Wurth S, Sader J, Cerutti B, et al.: Medical students' perceptions and coping strategies during the first wave of the COVID-19 pandemic: studies, clinical implication, and professional identity. BMC Med Educ. 2021; 21(1): 620). It is not relevant for the current study to describe the survey in detail again.  

 Reflexivity or Roles by Authors should be located at the end, rather than be a content in the main study text. We moved the Reflexivity section after the Consent to publish section.  

**Statistical analysis – OK**    

**Results**   Ideally the result should answer the aims of the study which are to determine 1) students’ performance, 2) self-assessment of performance and 3) perception of students towards the switching from summative to formative approach. This section ideally presented using table for the demographic data.  

 Please do include the disciplines during bachelor and master in the table format. We added a table in the Methods section after the second paragraph (Table 2).   A

 ll the numbers in this section will be best presented in table form, so that comparison between bachelor, masters, discipline, number of attempts can be made adequately, not in narrative form. All the results described in the Results section are reported as figures.     

 Table 1 should be in Result section. The data presented in Table 1 are not a result, but a description of the cohorts of students. This is the reason why Table 1 is in the Methods section.  

 My suggestion to review the usage of box plot in Figure 2, how best each discipline be presented and easily understood by the readers. Box plots are adequate to present the results of each formative assessment. They are visual and informative.  

 Major rephrasing and restructuring of this section are needed so that the readers able to appreciate the process and the flow of result of this study. The Results section is logically structured according to the aims of the study: sub section Formative assessments, which describes students’ performance (aim 1), and students’ self-assessment of performance (aim 2), followed by sub section Survey, which relates to students’ perception of the switch from summative to formative assessment (aim 3). The manuscript is written using the usual descriptive style.  

 For Figure 5, I believe that bar chart will be better than pie chart as these are free texts and many ideas can be obtained from 1 respondent hence 100% of the pie chart doesn’t represent the whole batch. We modified Figure 5.  

 Figure 2 and Table 2 are explaining the same outcomes The editorial team rightly requested that we add some participants’ quotes in a table. It is commonly done in qualitative research and supports the results presented in Figure 5.  

**Discussion**  

 This section was written not according to the flow or the aims of the study. The best way to present is to match with the flow of the result so that the readers will be able to appreciate the discussion and relate directly with the result section. The Discussion section is written following the structure of the Results section, which follows the aims of the study: discussion of the students’ performance (aim 1), of the students’ self-assessment of performance (aim 2), and of the students’ perception of the switch from summative to formative assessments (aim 3).  

 There are numbers and percentages in discussion section that should be stated in result section as the discussion section will explain the nature of multiple attempts by the students. All the numbers and percentages mentioned in the Discussion section are reported in the Results section.  

 Major rephrasing and restructuring of this section are needed so that the readers able to appreciate the flow of discussion of this study. As mentioned previously, the Discussion section is written following the structure of the Results section, which follows the aims of the study.  

 For limitation and strength, suggest starting with strength and followed by limitation. The author needs to show to the reader why this study is important and can be learnt by others, then followed by the limitations occurred during the study.  

 We changed the order starting with strengths followed by limitations: “This study has several strengths and limitations. The formative assessments offered to medical students of the Geneva Faculty of Medecine were robust: the questions were taken from the pool used for the summative examinations, students were given detailed feedback, and they had the opportunity to do the test again and monitor their progress. However, we do not have any information about the learning behaviour of the students who made only one attempt. They may have studied after the test to fill knowledge gaps and have benefited from the feedback offered. The participation in formative assessments was mandatory, so we do not know how the students would have behaved if the assessments had been optional. We observed a low participation rate in a couple of early formative assessments which were kept optional because of short decisional and organizational timelines. “    

**Conclusion**    

 The conclusion adequately portrait the summary of the study. Due to the missing students’ performance, the last sentence can’t be made as a conclusion, as the study at present showed self-perception of performance rather than actual performance (as the responses were anonymously recorded). We do present students’ actual performances (see Figures 1, 2, and 3).  

 Conclusion might need rewriting based on the new writeup of Result and Discussion sections. Since the purpose of the conclusion is to give clear and synthetic messages, we prefer not to change it.

# Reviewer report: Reviewer response for version 2

**DOI:** 10.21956/mep.21158.r34425

**Published:** 2023-08-14

**Authors:** Mohamad Rahal, Fouad Sakr

**Affiliations:** 1 School of Pharmacy, Lebanese International University, Beqaa Governorate, Beqaa Governorate, Lebanon

No further comments.

Have any limitations of the research been acknowledged?

Yes

Is the work clearly and accurately presented and does it cite the current literature?

Partly

If applicable, is the statistical analysis and its interpretation appropriate?

Yes

Are all the source data underlying the results available to ensure full reproducibility?

Yes

Is the study design appropriate and is the work technically sound?

Yes

Are the conclusions drawn adequately supported by the results?

Yes

Are sufficient details of methods and analysis provided to allow replication by others?

Yes

Reviewer Expertise:

Pharmacy and medical education; Learning and assessment; Pharmacoepidemiology; Pharmaceutical sciences

We confirm that we have read this submission and believe that we have an appropriate level of expertise to confirm that it is of an acceptable scientific standard.

# Reviewer report: Reviewer response for version 1

**DOI:** 10.21956/mep.20817.r34249

**Published:** 2023-10-12

**Authors:** Fernanda M Arantes-Costa

**Affiliations:** 1 CEDEM, School of Medicine of Universidade de São Paulo, São Paulo, Brazil

Thank you for inviting me to review this manuscript. The topic is intriguing, but the paper requires significant revisions before it can be considered for indexing. Below are my comments:

 The study investigated the impact of replacing summative examinations with mandatory formative assessments at the Faculty of Medicine, University of Geneva, in response to the COVID-19 pandemic. Summative exams were canceled, and students were required to participate in formative assessments, but passing was not mandatory. The study aimed to assess students' performance and their perception of the change.

 The online and distance formative assessments affected all medical students from years 2 to 5. Formative assessments were conducted online using a secure platform and closely resembled the usual exams in content and format. Students received detailed feedback after each attempt. A survey was conducted to gather students' perceptions of the switch to formative assessments.

 Most students (72.2%) did not repeat the test, missing the opportunity for increased learning. However, students who repeated the test showed improved scores, suggesting that retesting was beneficial.

 Students' self-assessment of scores was suboptimal, with high performers underestimating their scores and low performers overestimating them.

 Interestingly, most students did not seek additional support in learning, and there was no significant association between the wish for support and self-assessment or actual scores.

 Medical students generally supported the switch to formative assessments, primarily due to the stress alleviation during the pandemic.

 The study highlights that medical students welcomed the switch from summative to formative assessments during the pandemic. Nevertheless, a drawback was observed, as it led to decreased motivation to study, despite the mandatory formative assessments.

 Students who took the test multiple times showed improved performance, indicating the benefits of detailed feedback and retesting.

 The study also reveals that students' self-assessment of scores was inaccurate, with potential implications for their learning and performance.

 The manuscript presented is appropriate and covers an interesting aspect of the COVID-19 pandemic's impact on educational institutions. Implementing new strategies for teaching and assessing students during the pandemic, particularly using formative assessments as an alternative to traditional summative assessments, is highlighted. However, it is essential to acknowledge that this approach does not guarantee progress throughout the course. Moreover, students learn content and how to respond autonomously and in socially desirable ways.

 The study identifies two groups of students: those who use assessment opportunities to improve their learning and those who perform them out of obligation. Conducting a correlation analysis between these groups and various factors would enhance the quality of the data presented. Additionally, it is crucial to clarify how the repetition of assessments was conducted and to analyze Bachelor and Master year students separately. Students closer to medical practice may exhibit a greater tendency to take advantage of feedback and learning opportunities.

 Have you considered exploring learning styles as well? This could be an interesting topic, as students' perceptions of assessment models and feedback optimization are closely related to their learning styles. Additionally, delving into the differences between growth and fixed mindsets could be a valuable point of discussion. By exploring these two lines of inquiry, you may gain insight into how some students embrace challenges and take more risks, while others do not. Finally, investigating how motivation can be affected by a shift from summative to formative assessment could be an insightful addition to the conversation. Taking into account the mindset and learning style of each student might help in understanding why some students experience diminished motivation while others experience an increase in motivation.

Have any limitations of the research been acknowledged?

Yes

Is the work clearly and accurately presented and does it cite the current literature?

Partly

If applicable, is the statistical analysis and its interpretation appropriate?

Partly

Are all the source data underlying the results available to ensure full reproducibility?

Partly

Is the study design appropriate and is the work technically sound?

Yes

Are the conclusions drawn adequately supported by the results?

Partly

Are sufficient details of methods and analysis provided to allow replication by others?

Partly

Reviewer Expertise:

PhD in Sciences, Specialist in Medical Education, Coordinator of the Health Education Research Group at CEDEM in the Faculty of Medicine of USP. I study topics such as faculty development, assessment, healthcare student's quality of life, resilience, and happiness.

I confirm that I have read this submission and believe that I have an appropriate level of expertise to confirm that it is of an acceptable scientific standard, however I have significant reservations, as outlined above.

# Reviewer report: Reviewer response for version 1

**DOI:** 10.21956/mep.20817.r33080

**Published:** 2023-04-17

**Authors:** Mohamad Rahal, Fouad Sakr

**Affiliations:** 1 School of Pharmacy, Lebanese International University, Beqaa Governorate, Beqaa Governorate, Lebanon

Thank you for the invitation to review the paper “Formative assessments during COVID-19 pandemic: an observational study on performance and experiences of medical students”. It is an interesting topic but the paper can benefit from a major revision before being accepted for indexing.

 Here are my comments:

Introduction

- The Introduction should elaborate about summative and formative assessments among medical students in general.
- The first sentence of the Introduction could be referenced since it mentioned that many schools adapted changes and switched to online teaching.
- Please use the name of the Faculty of Medicine or its abbreviation throughout the text rather than referring to it as “our”.
- Please restructure and combine the following sentences in order to reflect that the information are from the same study “. In June 2020, an online survey was conducted among all the medical students from year 2 to 6 ( Wurth *et al.*, 2021). This study focused on students' main activities, the impact of the pandemic on their personal life, training and professional identity.”

Methods

- The Methods should start with a study design and setting. The design of the study should be mentioned clearly in the Methods, and which students were included in the analysis should also be clearly stated (all years or selected years) at the beginning of the methods. The first paragraph of the methods appears to be a program description rather than the inclusion criteria in the study.
- The courses/modules that the assessments took place in should be relocated to the study setting rather than the formative assessment section.
- When was the survey administered? One time after all assessments, or after each assessment? Please specify.
- The number of possible attempts appears confusing in the bachelor years.

Results

- The results should start with sample description, number of students in each year, courses, etc., because they were used later in the analyses.
- Why were the survey responses made by 6 ^th^ year students not coded?
- In Figure 2, the number of attempts are missing for primary care, psychiatry, and surgery.
- Why the authors didn’t correlate students’ perception and assessment outcomes? An additional analysis modeling students’ perception category (from the survey) and formative assessment outcomes would be very important.

Discussion

- The authors have to discuss more why there was a significantly higher single attempts by bachelor students compared to master’s students. The difference between fundamental knowledge and clinical usefulness of topics does not appear to be a valid reason.
- More details about the questions in the second and third attempts should be discussed. Did the assessment involve the same or different questions in each attempt? Or what percentage of questions from the first attempt is repeated in the second and third attempt?
- Around 40% of participants didn’t provide their feedback through the survey assessment - how this can affect the generalizability of the findings about students’ perception?
- The referencing style should be unified throughout the manuscript, for example, the authors used citation number [10] in one paragraph of the Discussion whilst they used the APA style (author and year) throughout most of the text.
- The self-assessment of scores comparison would have been better to be compared between academic years, courses/modules rather than gender. What are pedagogical outcomes from the gender comparison?
- Participation in multiple assessment attempts does not necessarily correlate with better knowledge and clinical work because the average time between attempts was less than 24 hours. This time does not seem adequate to have a greater learning opportunity as mentioned by the authors.
- The discussion should include practical implications for the findings of the study. This discussion part should be inserted before the limitations.

Conclusion:

- The conclusion should reflect the results and data of the findings rather than hypothesis. So the following sentence can be hypothesized and presented in the discussion rather than a final conclusion: “Students who did take the test more than once significantly improved their performance, which supports a benefit of the combination of detailed feedback, learning time, and re-test opportunity”. The authors should not include in the conclusion the reason for students’ performance enhancements.

Have any limitations of the research been acknowledged?

Yes

Is the work clearly and accurately presented and does it cite the current literature?

Partly

If applicable, is the statistical analysis and its interpretation appropriate?

Yes

Are all the source data underlying the results available to ensure full reproducibility?

Yes

Is the study design appropriate and is the work technically sound?

Yes

Are the conclusions drawn adequately supported by the results?

Yes

Are sufficient details of methods and analysis provided to allow replication by others?

Yes

Reviewer Expertise:

Pharmacy and medical education; Learning and assessment; Pharmacoepidemiology; Pharmaceutical sciences

We confirm that we have read this submission and believe that we have an appropriate level of expertise to confirm that it is of an acceptable scientific standard, however we have significant reservations, as outlined above.

# Response

**Published:** 2023-07-27

**Authors:** Vanessa Lavallard

**Affiliations:** Faculty of Medicine of Geneva, Switzerland

We thank the reviewers for their insightful feedback and comments on our manuscript" Formative assessments during COVID-19 pandemic: an observational study on performance and experiences of medical students", which helped us improve it. Please find our answers below. 

Introduction

- The Introduction should elaborate about summative and formative assessments among medical students in general.
  We gave a more detailed description of summative and formative assessments in the Introduction (second paragraph):

“The main aim of summative assessment is to evaluate whether the required skills and knowledge have been acquired at some definite timepoint. Assessments are usually high-stakes as they are used for promotion decisions. Formative assessment is a process of evaluation and feedback. The objective is to support and accompany the learning process. Formative assessments are typically low-stakes, ongoing, and seek to determine where the students are in their learning, what they need to improve and how they can do it.”  

- The first sentence of the Introduction could be referenced since it mentioned that many schools adapted changes and switched to online teaching.

We added three references in the Introduction regarding the adaptations made by medical schools on teaching during the COVID-19 pandemic: Dost et al. 2020; https://doi.org/10.1136/bmjopen-2020-042378 Stoehr et al. 2021; https://doi.org/10.1371/journal.pone.0257394 LeBlanc III 2022) ; https://doi.org/https://doi.org/10.18103/mra.v10i9.3081.

  

- Please use the name of the Faculty of Medicine or its abbreviation throughout the text rather than referring to it as “our”.

Done.  

- Please restructure and combine the following sentences in order to reflect that the information are from the same study “. In June 2020, an online survey was conducted among all the medical students from year 2 to 6 ( Wurth *et al.*, 2021). This study focused on students' main activities, the impact of the pandemic on their personal life, training and professional identity.”

We modified the text as suggested: “In June 2020, an online survey was conducted among all the medical students from Year 2 to 6 to determine how students were organizing their activities, and the impact of the pandemic on their personal life, training, and professional identity (Wurth et al., 2021).” 

Methods

- The Methods should start with a study design and setting. The design of the study should be mentioned clearly in the Methods, and which students were included in the analysis should also be clearly stated (all years or selected years) at the beginning of the methods. The first paragraph of the methods appears to be a program description rather than the inclusion criteria in the study.

We revised the Methods section to clarify the design of the study at the beginning. The Table 1 gives the distribution of students by year and gender.

  

- The courses/modules that the assessments took place in should be relocated to the study setting rather than the formative assessment section.

We did as suggested.

  

- When was the survey administered? One time after all assessments, or after each assessment? Please specify.
  The survey was a one–shot cross sectional survey conducted among all the students in June 2020. Hence, it was not administered after every assessment.

- The number of possible attempts appears confusing in the bachelor years.

Three written formative assessments (namely respiration, osteo-articular, and infectious diseases) allowed two attempts (one attempt per 24-hour period). Two written formative assessments (namely integration and community dimensions) allowed three attempts within a five-day period. We modified the text to make it clearer. 

Results

- The results should start with sample description, number of students in each year, courses, etc., because they were used later in the analyses.

We described the number and types of courses in Bachelor and Master years and the table 1 gives the distribution of students by year and gender.

  

- Why were the survey responses made by 6 ^th^ year students not coded?
  The sixth and last year of the pre-graduate curriculum is entirely dedicated to clinical clerkships. There are no faculty summative exams, so there was no switch to any formative assessments. This is the reason why we did not analyse the answers of these students.

 

- In Figure 2, the number of attempts are missing for primary care, psychiatry, and surgery.

We thank the reviewers for pointing out this oversight. Figure 2 was corrected.

  

- Why the authors didn’t correlate students’ perception and assessment outcomes? An additional analysis modeling students’ perception category (from the survey) and formative assessment outcomes would be very important.

The survey was anonymous, we were not able to estimate any correlation. The survey and our study were originally designed as separate studies. 

Discussion

- The authors have to discuss more why there was a significantly higher single attempts by bachelor students compared to master’s students. The difference between fundamental knowledge and clinical usefulness of topics does not appear to be a valid reason.

The students’ comments in the survey show that students tended to study what they thought useful for clinical practice, and left out other, less related topics. Students were less motivated to study than for summative assessments. Taken together, these elements support our hypothesis. This is a novel finding; we found no data in the literature about this topic. Therefore, we prefer not to discuss it further.

  

- More details about the questions in the second and third attempts should be discussed. Did the assessment involve the same or different questions in each attempt? Or what percentage of questions from the first attempt is repeated in the second and third attempt?

The assessment in each discipline involved exactly the same set of questions for each attempt. We added a sentence in the Methods section, sub-section Formative assessment: ”The same set of questions was used for each attempt.»

  

- Around 40% of participants didn’t provide their feedback through the survey assessment - how this can affect the generalizability of the findings about students’ perception?
  The analysis of the survey was qualitative and focused on identifying themes, which is not dependent on the sheer number of respondents. We cannot exclude that some themes would have emerged if more students had answered. However, it is unlikely because we found a broad homogeneity in the answers. In previous surveys, a 50% response rate allowed us to obtain reliable results regarding a whole cohort’s perception. Previous generalizability studies conducted in our institution estimated that, considering the size of our cohorts of students, a 50% response rate would be compliant with the capacity to keep the standard error of the mean within the limits of desired levels of precision.  

*Reference*: Gerbase MW, Germond M, Cerutti B, Vu NV, Baroffio A. How Many Responses Do We Need? Using Generalizability Analysis to Estimate Minimum Necessary Response Rates for Online Student Evaluations. Teach Learn Med. 2015;27(4):395-403. doi: 10.1080/10401334.2015.1077126. PMID: 26507997.

- The referencing style should be unified throughout the manuscript, for example, the authors used citation number [10] in one paragraph of the Discussion whilst they used the APA style (author and year) throughout most of the text.
  Thanks for pointing that out.  We have corrected the referencing style.

 

- The self-assessment of scores comparison would have been better to be compared between academic years, courses/modules rather than gender. What are pedagogical outcomes from the gender comparison?
  Many studies have dealt with gender-group differences in learning behaviour, hence it was relevant to split our results by gender. In addition, desegregation of data by gender is often promoted and recommended.

*References: * Colaço R, Watson-Grant S. A Global Call to Action for Gender-Inclusive Data Collection and Use. 2021 Dec. In: RTI Press Policy Brief [Internet]. Research Triangle Park (NC): RTI Press; 2010–. PMID: 36862818.   Newton JN, Griffiths C, Fitzpatrick J, Lamagni T, Campos-Matos I. Sex-disaggregated data is reported by Public Health England. Lancet Glob Health. 2021 Aug;9(8):e1059. doi: 10.1016/S2214-109X(21)00249-7. Epub 2021 Jun 23. PMID: 34174186; PMCID: PMC8443002. We also made some comparisons (not reported) by academic years: Master students clearly underestimated their performance more frequently than Bachelor students did (see the figure in supplementary data: https://doi.org/10.6084/m9.figshare.21755882) We agree with the reviewers that pedagogical outcomes are an important issue. To draw attention to biases – gender or other biases – matters, but may not be sufficient to change attitudes. An analysis of the clinical vignettes developed for teaching at the Geneva Faculty of Medicine showed that gender professional roles were stereotyped. Physicians were often male, while nowadays most medical students and more and more physicians are female. A similar observation was made with regards to the scenarios for the clinical skills examination (OSCEs) of the Swiss federal licensing exam. Our results support the change in learning material about to be implemented in the Geneva Faculty of Medicine. To depict women in leading positions will strengthen the image of women as competent professionals. It also better reflects the actual situation since more and more women occupy leading and academic positions. It would be interesting to determine whether female undergraduate medical students still underestimate their performance more than male students in a few years’ time. We added a comment in the Discussion: “The difference can also be related to gender bias. In the clinical vignettes used for teaching at the Geneva Faculty of Medicine gender professional roles tend to be stereotypes, e.g. physicians are more often male. To depict women in leading positions would strengthen the image of women as competent professionals.»  

- Participation in multiple assessment attempts does not necessarily correlate with better knowledge and clinical work because the average time between attempts was less than 24 hours. This time does not seem adequate to have a greater learning opportunity as mentioned by the authors.
  We would agree with this comment if the students had to answer different questions at the second and third attempts. Since all the attempts involved the same set of questions, the students had enough time to study what they did not know.

- The discussion should include practical implications for the findings of the study. This discussion part should be inserted before the limitations.

Thank you for suggesting this addition. Our results support changes in the teaching material concerning the socio-professional characteristics of women (see above): “To depict women in leading positions would strengthen the image of women as competent professionals”. We added a paragraph in the Discussion section before the study limitations that describes other practical implications: “Our findings underscore the need for formative assessment to be part of a larger assessment system (programmatic assessment) to motivate and benefit medical students best. The role of scholar defined in the CanMEDS (https://www.royalcollege.ca/ca/en/canmeds/canmeds-framework.html) or PROFILES (https://www.profilesmed.ch) frameworks requires lifelong learning and a planned approach to learning from physicians. In a competency-based curriculum, students must be supported to acquire the expected autonomy in self-improvement. Regular self-evaluation of scores at formative and summative assessments could help medical students better estimate their competences. Overestimation of performance is a marker for potentially less competent students. Recurrent overestimations could be used to identify medical students in need of support in learning.” 

Conclusion:

- The conclusion should reflect the results and data of the findings rather than hypothesis. So the following sentence can be hypothesized and presented in the discussion rather than a final conclusion: “Students who did take the test more than once significantly improved their performance, which supports a benefit of the combination of detailed feedback, learning time, and re-test opportunity”. The authors should not include in the conclusion the reason for students’ performance enhancements.

As suggested, we moved the following sentence to the beginning of the discussion: «[Among the 30.8% of examinees who repeated the test, the average score increased, and the average duration of the attempt decreased], which supports a benefit of the combination of detailed feedback, learning time, and re-test opportunity ».
